# Design and optimization of a transparent and flexible MIMO antenna for compact IoT and 5G applications

**DOI:** 10.1038/s41598-023-47458-1

**Published:** 2023-11-23

**Authors:** Muhammad Nawaz Abbasi, Abdul Aziz, Khaled AlJaloud, Abdul Rehman Chishti, Ali H. Alqahtani, Durria Abbasi, Farooq A. Tahir, Zia Ullah Khan, Rifaqat Hussain

**Affiliations:** 1 Faculty of Engineering & Technology, The Islamia University of Bahawalpur, Bahawalpur, Pakistan; 2 College of Engineering, Muzahimiyah Branch, King Saud University, P.O. Box 2454, Riyadh, 11451 Saudi Arabia; 3 School of Electrical Engineering and Computer Science, National University of Sciences and Technology (NUST), Islamabad, Pakistan; 4 Department of Electronic and Electrical Engineering, The University of Sheffield, Sheffield, UK; 5 Antenna and Electromagnetics Research Group, School of Electronic Engineering and Computer Science, Queen Mary University of London, London, UK

**Keywords:** Electrical and electronic engineering, Aerospace engineering

## Abstract

This work presents an optically transparent and flexible MIMO antenna that features two square patch elements placed in close proximity, aiming to meet the demands of compactness, flexibility, optical transparency, and visual appeal for IoT applications and future 5G wireless communication. The design includes a simple offset fed configuration to achieve the required isolation and impedance matching. It simplifies the process of creating closely spaced transparent MIMO antenna configurations. By optimizing and analyzing this structure, the antenna achieves better isolation and diversity gain performance, even when the patch elements are positioned very close to each other. To achieve optical transparency and flexibility, the antenna uses thin polyethylene terephthalate (PET) material as a substrate, which is a thermoplastic polymer resin from the polyester family. The wired metal mesh parameters for conducting parts of the MIMO antenna and offset position of the feed are carefully optimized to achieve required optical transparency, isolation, impedance matching and radiation performance without any complex decoupling or impedance matching network.

## Introduction

Multi-input-multi-output (MIMO) antenna systems are used to enhance spectral efficiency and channel capacities of 5G and IoT infrastructure^1,2^. Transparent and flexible MIMO antennas serve as a key element to maintain compatibility with devices of varying shapes and sizes while minimizing their visual impact. The optical transparency may help to increase visual aesthetics of 5G-enabled base stations, smartphones, tablets and wide range of IoT devices, from environmental sensors to home automation devices. While the flexibility of the antenna design might make it suitable for integration into flexible communication modules or boards that can be adapted to various types of IoT sensor nodes and devices with different form factor.

These can also help to install more antennas in close proximity to enhance signal strength and data speeds^3^ for next-generation compact, transparent, and flexible 5G systems. However, a transparent and flexible MIMO antenna configuration is challenging to realize due to complex isolation techniques, such as vias and decoupling structures, which are not ideal for maintaining transparency and low-cost manufacturing processes^4,5^.

During the past few years, State-of-the-art flexible and transparent MIMO antennas have been developed using advanced nanotechnology. Researchers have explored various conductive and optically transparent materials, including AgHTs (silver-coated polymers), IZTO (indium zinc thin oxide), ITO (indium tin oxide), and graphene, for their potential use in the development of transparent and flexible antennas for wearable applications. However, AgHTs suffer from high surface resistance and low radiation efficiency for antennas^6^ ,^7^ despite having good flexibility. Furthermore, IZTO and ITO materials are not suitable for future transparent and flexible applications due to their brittleness and high levels of loss when bent, as referenced in^8^. Roll-to-roll production of monolayer graphene has been successfully demonstrated, boasting high transparency, electron mobility, and scalability. However, the transfer of large, high-quality graphene films remains a significant challenge, as mentioned in^9^ and^10^.

The use of metallic mesh (MM) has been viewed as a promising technique for transparent and flexible applications owing to its superior transparency, conductivity, and malleability. The full potential of MM structures in contemporary radio frequency devices has not been completely investigated yet. In general, micro-structured MM films have found applications in solar cells, electromagnetic interference shielding^11^, and all-solid-state supercapacitors^12^. However, there are only a few reported instances of stiff antennas using MMs as conductive electrodes in RF devices. There were reports of low efficiency and poor transparency in these MM antennas, as indicated in^13^ and^14^.

In^15^, transparent and flexible antennas are proposed, which offer good performance through the use of metallic mesh with embedded Nickel as conductive electrodes using a selective electrode position process in combination with inverted film-processing on polyethylene terephthalate (PET) substrates. A 4-element transparent flexible MIMO is presented for sub-6 GHz 5G applications in^16^. The researchers conducted parametric studies on antenna resonator geometry to ensure minimum fabrication errors, while maintaining high isolation levels across wide operation bandwidth. However, it is realized by carefully designing the connected ground plane lines and introducing complex slots in the substrate. Thus, developing flexible antennas with exceptional transparency and optimum efficiency is a challenging task. So, there is still need to achieve a simple and suitable solution for a transparent and flexible MIMO antenna configuration without using any decoupling structure between antenna elements, a defected ground structure or vias.

In this study, a simple, transparent and flexible MIMO antenna with two close proximity patch antenna elements is proposed to achieve higher isolation without using any decoupling structure or complex matching network. The frequency band of the proposed MIMO antenna is centered at 4.5 GHz, which falls within the frequency range considered suitable for 5G New Radio (NR) sub-6 GHz band and IoT applications. An optically transparent square lattice of wired metal mesh is printed on a flexible transparent polyethylene terephthalate (PET) substrate to achieve transparency. A simple offset fed configuration is used to achieve higher isolation and impedance matching for the closely spaced patch antenna elements. The proposed transparent and flexible MIMO antenna is analyzed and optimized through parametric analysis and surface current distribution analysis of the square lattice of the wired metal mesh. The desired optical transparency, impedance matching, isolation, radiation performance, and diversity gain performance with flexibility are achieved and these are also verified through measurement of the fabricated prototype.

## Design of flexible and transparent MIMO antenna

To evaluate the effectiveness of a transparent MIMO antenna utilizing a wired metal mesh, a MIMO antenna operating in the 4.5 GHz band is chosen, with two patch elements located in close proximity to each other.

**Figure 1:**
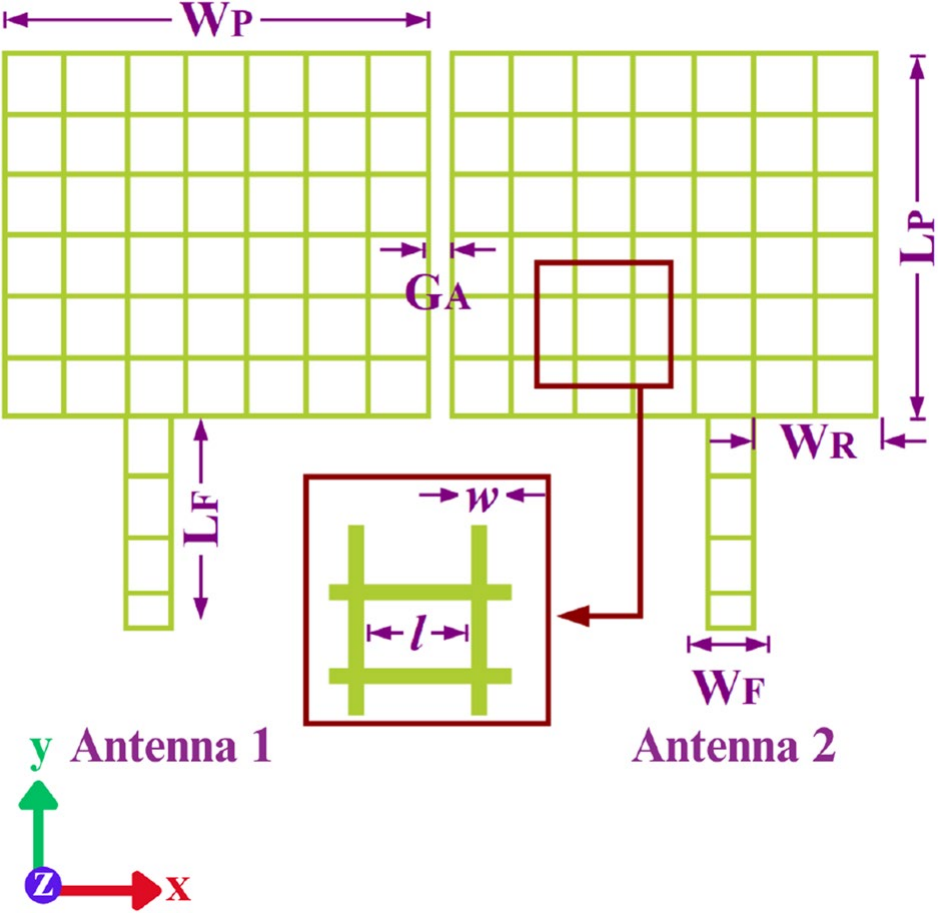
The proposed transparent antenna geometry with close proximity patch antenna elements.

Figure 1 illustrates the geometry of the proposed transparent and flexible MIMO antenna that consists of two square patch antenna elements positioned in close proximity, with 0.9 mm ($0.0135 \times \lambda$ at 4.5 GHz) spacing. To ensure optical transparency, a wired metal mesh with a square lattice is used, while the substrate material is 0.1 mm thick optically transparent PET material. The PET substrate has a dielectric constant of 3.5.

No decoupling or matching network is used to achieve isolation or impedance matching respectively, for the MIMO antenna configuration. Instead, a simple offset edge fed is used for excitation of both patch antenna elements. This simple asymmetric edge fed configuration is also suitable for development of large antenna arrays. The wired metal mesh square lattice is used in the proposed antenna to achieve approximately 83% optical transparency, with size of the square lattice denoted as $'l'$ and the wire width as $'w'$. The design process, analysis, and optimization of the proposed geometry are performed using CST Microwave Studio. The optimized dimensions for the proposed geometry can be found in Table 1

**Table 1:** Parameters of the proposed MIMO antenna.

Parameters	*l*	*w*	$W_P$	$L_P$	$W_F$	$L_F$	$W_R$	$G_A$
Values (mm)	2	0.2	16	18	2	7.75	3.5	0.9

**Figure 2:**
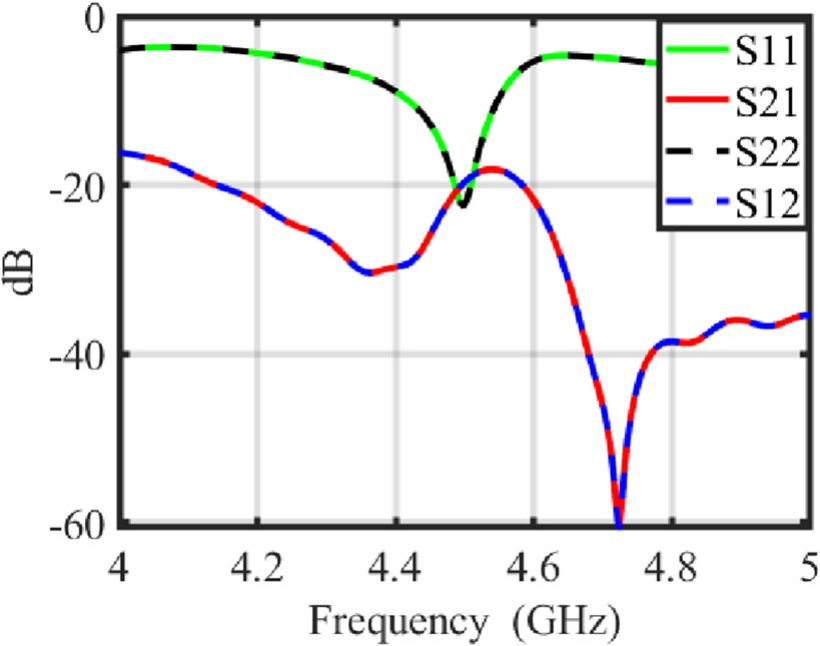
Scattering parameters of the proposed transparent and flexible MIMO antenna.

Figure 2 displays the scattering parameters of the proposed transparent and flexible MIMO antenna. Due to symmetric configuration of both the antenna elements around y-axis, the S12 curve overlaps with the S21 curve. Similarly, the S11 and S22 curves also overlap. The S11 and S22 curves demonstrate that the proposed transparent MIMO antenna exhibits excellent impedance matching at 4.5 GHz. Moreover, the isolation between the closely spaced transparent elements exceeds 19 dB in the desired frequency band, indicating that the proposed transparent and flexible MIMO antenna has potential to achieve better diversity gain performance as well as good impedance matching without using any complex decoupling or matching network.

### Role of offset feed configuration to achieve higher isolation

Generally, when two antennas are placed in close proximity to each other, these tend to couple or interact with each other, which can lead to poor isolation between the antennas. To overcome this issue, some decoupling structures and techniques are usually employed in literature to achieve better isolation. However, a decoupling structure between antenna elements may cause larger separation between antennas and may also increase power loss, which may limit compactness of MIMO antenna and may also reduce radiation efficiency.

In this work, the offset edge fed plays a crucial role in achieving higher isolation between the patch antenna elements and good impedance matching.The fed point for both of the antennas is intentionally shifted or offset from the center of the antenna elements. By optimizing the offset position of the edge fed, higher isolation between the closely spaced patch elements as well as required impedance matching for each antenna and feed line can be achieved.

**Figure 3:**
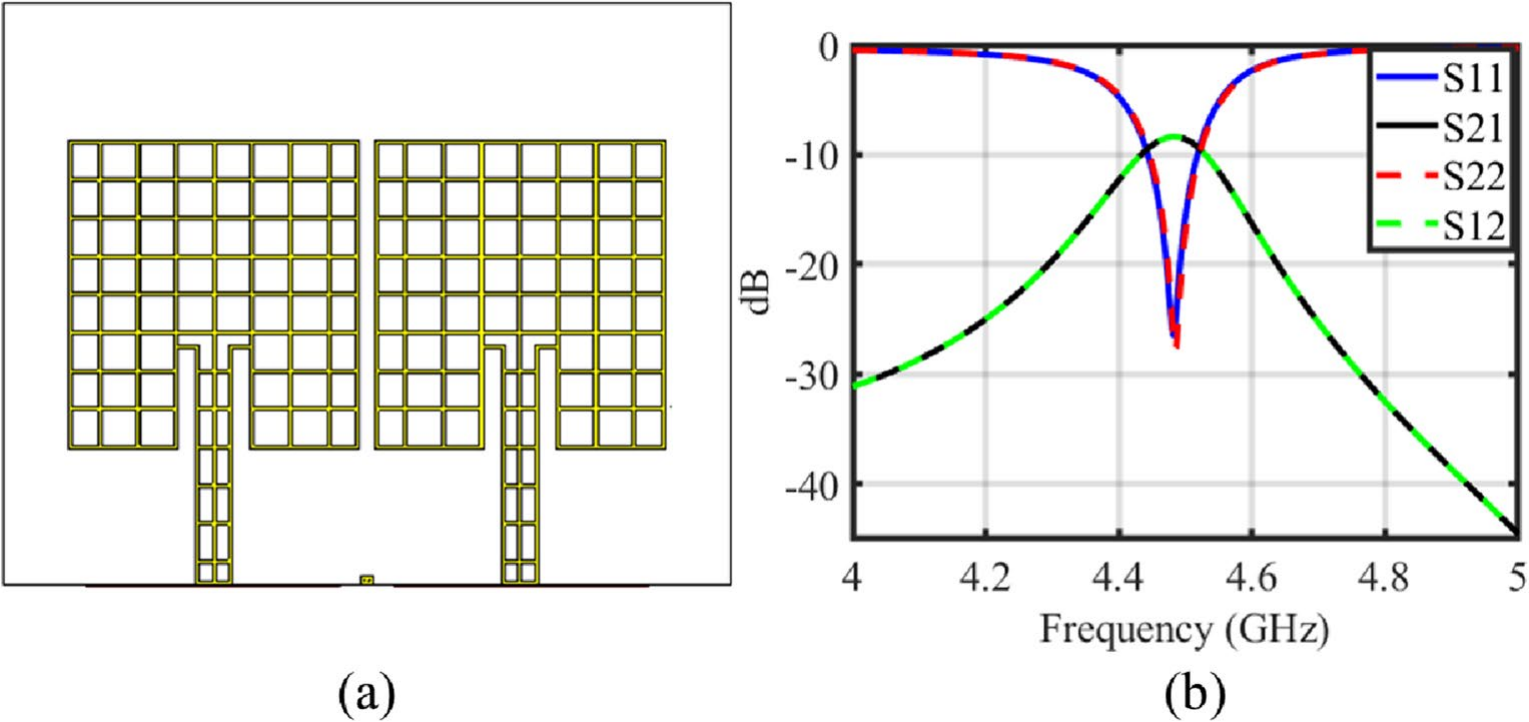
Conventional center inset fed transparent and flexible MIMO antenna (**a**) geometry (**b**) scattering parameters.

**Figure 4:**
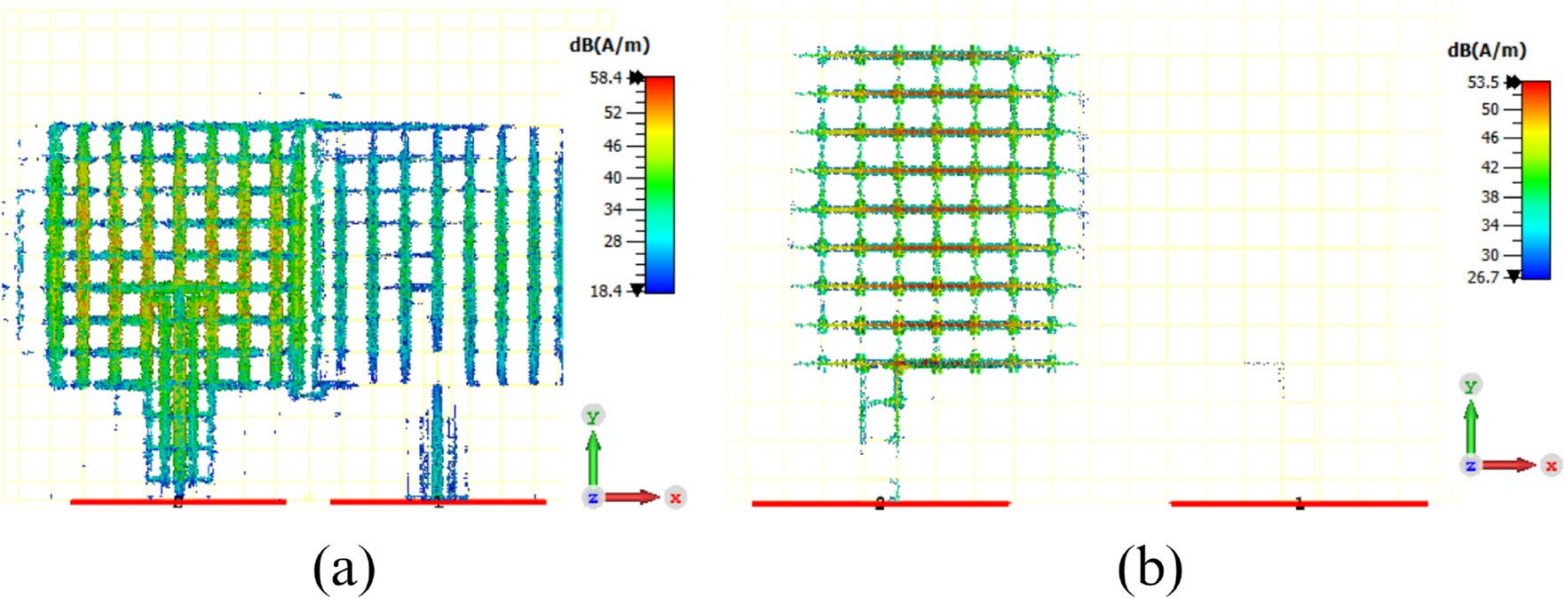
(**a**) Surface current distribution at 4.5 GHz for conventional centre fed MIMO antenna when antenna-1 is excited and antenna-2 is matched to 50$\Omega$ load. (**b**)Surface current distribution of the proposed transparent and flexible MIMO antenna at 4.5 GHz, when antenna-1 is excited and antenna-2 is matched to 50$\Omega$ load.

A center fed conventional MIMO antenna configuration with same geometrical parameters and spacing between antenna elements as those of the proposed MIMO antenna is also analyzed to verify reason for enhanced isolation due to offset fed configuration and shown in Fig. 3. Scattering parameters for the center fed conventional MIMO antenna are shown in Fig. 4b. It can be seen that the isolation for conventional center fed configuration is only 8 dB. The surface current distribution for conventional center fed MIMO antenna is also shown in Fig. 4a. It can be seen that when antenna-1 is excited and antenna-2 is matched to a 50$\Omega$ impedance, a symmetric current distribution is observed on antenna-1 due to center fed excitation. However a significant surface current coupling from antenna-1 to antenna-2 can also be observed, which confirms poor isolation performance for the center-fed configuration.

The offset feding is used to control current distribution on each antenna element of MIMO antenna, thereby improving isolation without using any decoupling structure between antenna elements. Figure 4b displays the surface current distribution of the MIMO antenna, which demonstrates enhanced isolation between antenna elements. The figure indicates that when antenna-1 is excited, there is almost no surface current coupling observed towards antenna-2.

It can be seen that the offset fed configuration causes phased current distribution on each antenna element and the current distribution is asymmetric and little more concentrated towards offset fed point of antenna-1, and there is almost no current is seen on antenna-2. Similar but mirror phased current distribution is also observed on other antenna element when antenna-2 is excited, which is not shown here for brevity. The asymmetric current distribution in one antenna phased in opposite direction to that of the other antenna, which reduces the mutual coupling between closely spaced antenna elements. The degree of decoupling achieved through offset feeding depends on various parameters, including the offset distance, antenna geometry, and frequency of operation.

**Figure 5:**
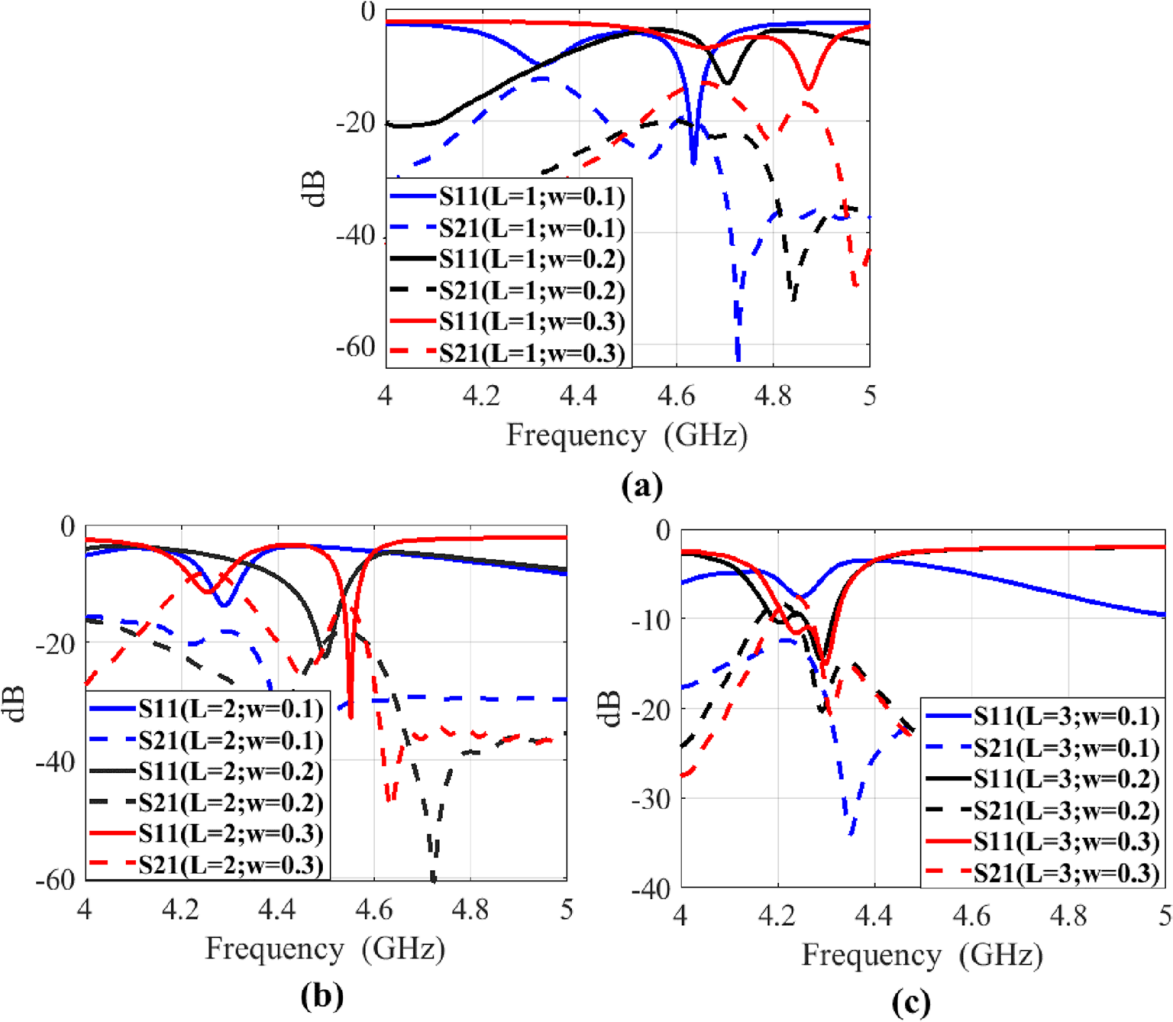
Parametric analysis for metal mesh lattice size of the proposed transparent and flexible MIMO antenna (**a**) $l=1~mm, ~w=0.1~to~0.3\,$mm, (**b**) $l=2$ mm,  $w=$0.1 to 0.3 mm, (**c**) $l=3$ mm,  $w=$0.1 to 0.3 mm.

**Table 2:** Parametric analysis of the MIMO antenna.

*l* (mm)	*w* (mm)	O.T (%)	Frequency (GHz)	$S_{11}$ (dB)	$S_{12}$ (dB)
1	0.1	83	4.63	− 28	− 19.5
1	0.2	69	4.72	− 11	− 24
1	0.3	59	4.88	− 15	− 17
2	0.1	91	4.27	− 12	− 20
2	0.2	83	4.5	− 22	− 19
2	0.3	76	4.55	− 34	− 16
3	0.1	94	4.26	− 7	− 13
3	0.2	88	4.28	− 15	− 10
3	0.3	83	4.3	− 15	− 11

## Optimization steps

Achieving optimal performance for multiple parameters simultaneously is a formidable task. Notably, there is still no significant work available in literature that is simultaneously focused to achieve desired optical transparency and flexibility for a MIMO antenna with closely spaced antenna elements. Optimization to achieve required transparency, flexibility, resonance frequency, impedance matching, isolation and radiation performance simultaneously, is a distinct and complex challenge because these performance metrics varies with the variation of the parameters of the square lattice of the wired metal mesh to achieve desired transparency as well.

In Fig. 5, parametric analysis is presented to investigate the effect of the square lattice dimensions $'w'$ and $'l'$ of a wired metal mesh on optical transparency, resonance frequency, impedance matching (S11) and isolation (S21). The parametric analysis performance is also summarized in Table 2. The purpose of this analysis is to demonstrate optimization steps for square lattice dimensions of the wired metal mesh to optimize performance of a MIMO antenna with closely spaced antenna elements, while maintaining suitable optical transparency and flexibility.

### Optical transparency

The definition of optical transparency (O.T) for square lattice of a wired metal mesh is given in^13^:

1 $$\begin{aligned} O.T= \left[ \frac{l}{ w + l} \right] ^2 \end{aligned}$$

Equation (1) reveals that O.T of a transparent antenna can be enhanced by increasing $'l'$ and decreasing $'w'$ due to decrease in coverage of copper, which may reduce radiation performance of antenna. So, balancing $'w'$ and $'l'$ to maximize O.T without sacrificing overall MIMO antenna performance with closely spaced antenna elements is a challenging task.

### Resonance frequency

Altering $'w'$ and $'l'$ values also affects the resonance frequency of each antenna element. Precision control of these parameters is necessary to achieve desired resonance frequency. Precisely managing $'w'$ and $'l'$ for accurate resonance frequency control is another challenge. It can be observed that increasing $'l'$ can lower the resonance frequency, while increasing $'w'$ may raise it. So, striking the right balance is crucial.

### Impedance matching

The $'w'$ and $'l'$ parameters also directly impacts impedance matching (S11 and S22) of each antenna element. It can also be observed that impedance matching of MIMO antenna elements varies randomly with variation of square lattice parameters. So, careful $'w'$ and $'l'$ adjustments are also needed to achieve proper impedance matching as well as required optical transparency.

### Isolation

The $'w'$ and $'l'$ adjustments also influence the coupling between antenna elements. Sensitivity in $'w'$ and $'l'$ is required to optimize isolation. It can be derived in general from above parametric analysis that the isolation (S21 and S12) between antenna elements decreases with increase in $'w'$ and $'l'$. So, careful fine tuning of these parameters also needed to achieve desired isolation as well as other performance metrics.

After this thorough analysis, we collectively determine the optimized values of $'w'$ and $'l'$. The optimal dimensions of $l = 2$ mm and *w* = 0.2 mm, are selected for the square lattice to achieve optimum optical transparency of 83%, return loss of 22 dB, and isolation of 19 dB at 4.5 GHz. These optimized values strike a balance between transparency, resonance frequency, impedance matching and isolation for transparent and flexible MIMO antenna with closely spaced antenna elements.

## Performance analysis

**Figure 6:**
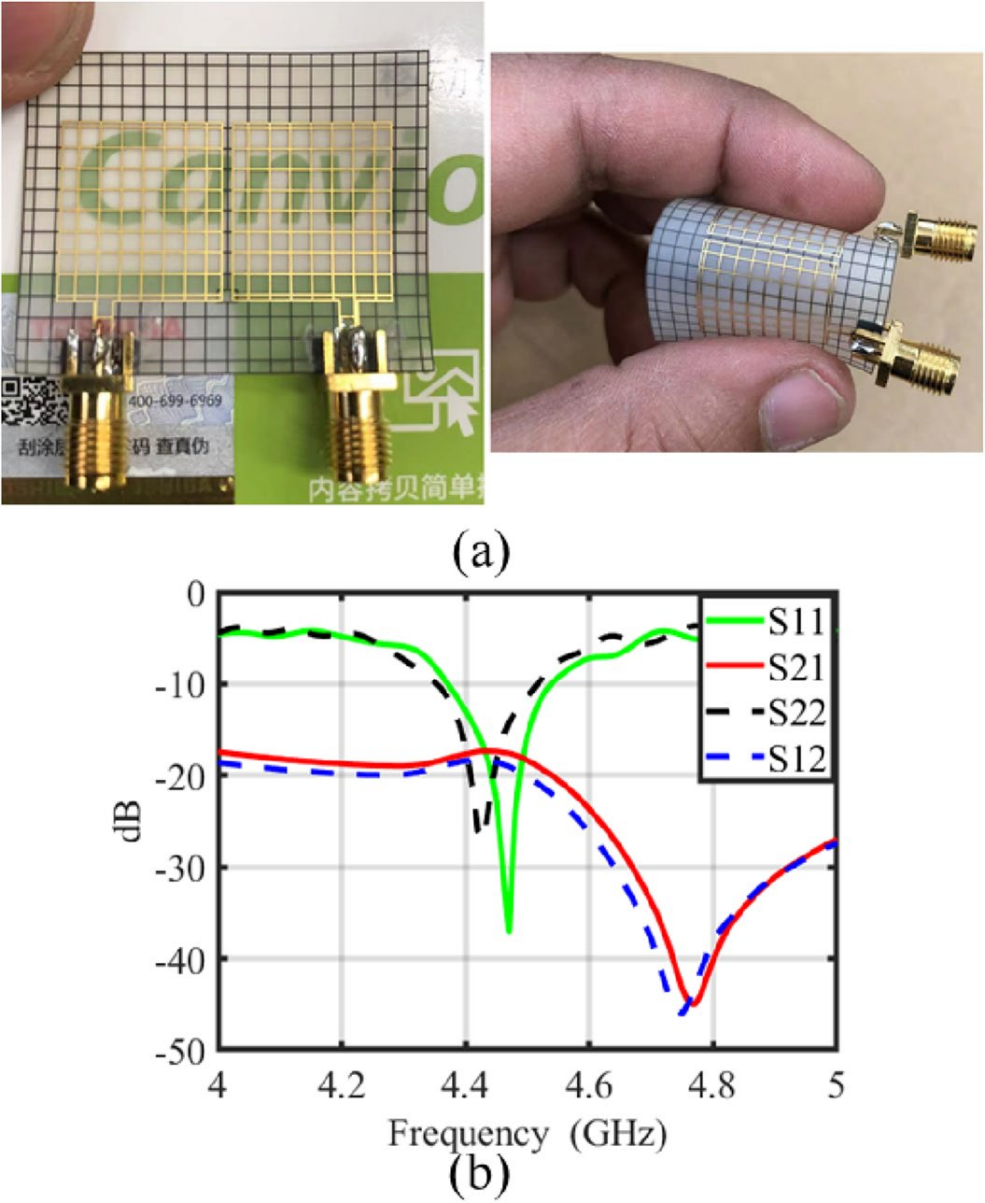
(**a**) Fabricated prototype of the proposed transparent MIMO antenna, (**b**) measured scattering parameters for the proposed transparent and flexible MIMO antenna.

The proposed transparent and flexible MIMO antenna has also been manufactured and tested to verify its simulated performance. Figure 6a shows the fabricated prototype, and Fig. 6b displays the measured scattering parameters. The results indicate that the simulated and measured scattering parameters are in close agreement. However, there are some slight deviations in the resonance frequency and transmission coefficient curves of both antenna elements. These discrepancies may be due to practical tolerance in simulation and measurement environments, as well as imperfect soldering connections.

**Figure 7:**
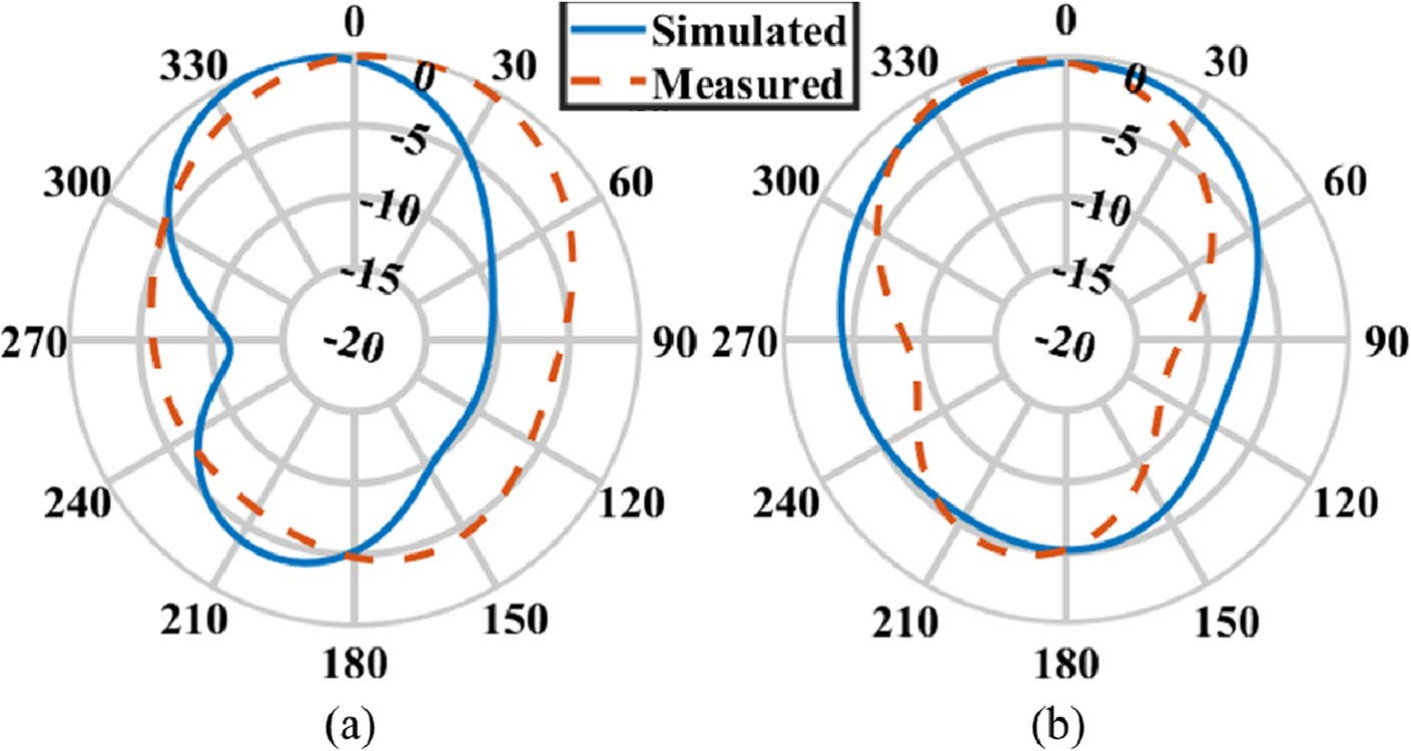
Radiation pattern of the transparent MIMO antenna at 4.5 GHz (**a**) E-plane, (**b**) H-plane.

**Figure 8:**
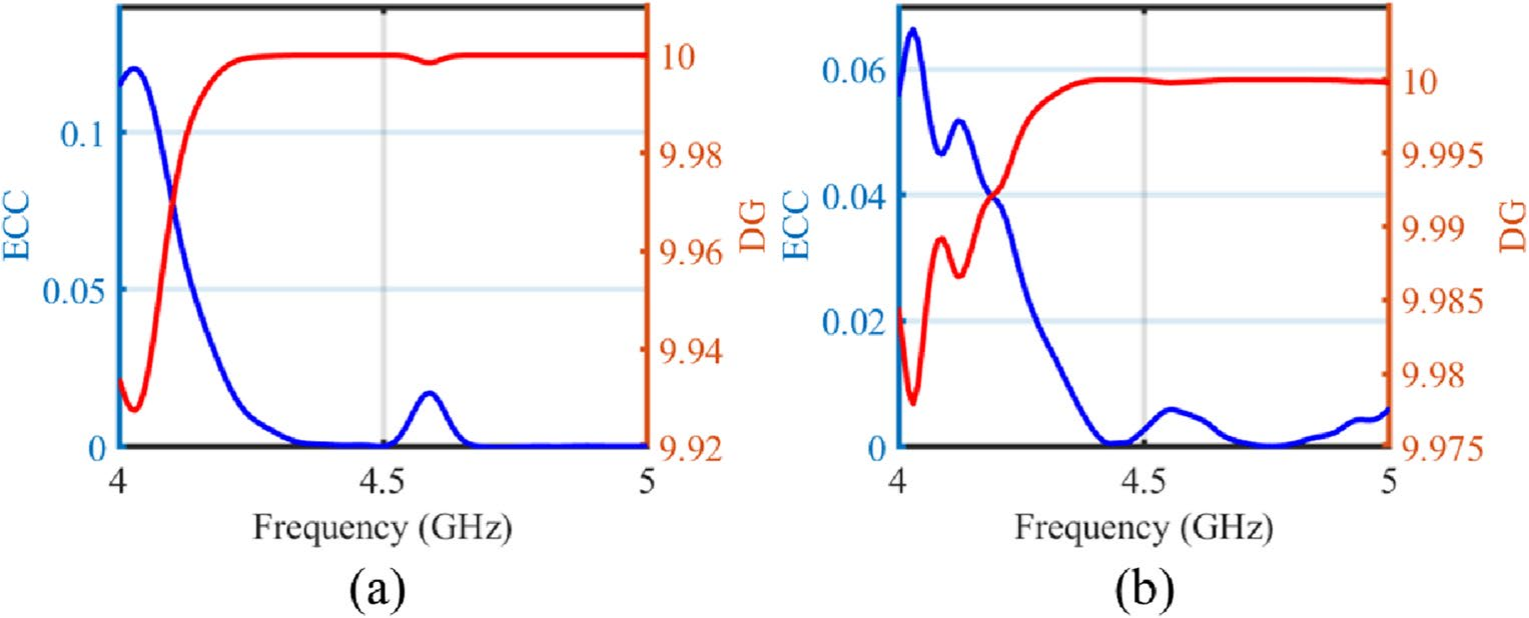
The ECC and DG performance of the transparent and flexible MIMO antenna (**a**) simulated, (**b**) measured.

In addition to the previous analysis, the proposed transparent MIMO antenna is evaluated for its performance using measures such as radiation pattern, Envelope Correlation Coefficient (ECC), Channel Capacity Loss (CCL), Total Active Reflection Coefficient, and Mean Effective Gain (MEG).

### Radiation pattern

Figure 7 displays the simulated and measured radiation patterns in both orthogonal planes at 4.5 GHz for one of the elements of the transparent patch antenna.

The transparent patch antenna element demonstrates good radiation performance at 4.5 GHz, with a gain of 3.2 dB, a radiation efficiency of 61%, and total efficiency of 63%. This is quite impressive for a transparent and flexible antenna element. The $2^{nd}$ closely spaced element also exhibits similar radiation performance.

#### Envelope correlation coefficient (ECC) and diversity gain (DG)

Envelope Correlation Coefficient (ECC) is utilized to assess the effect of coupling between antenna elements, measuring the correlation between them in a MIMO diversity system. Higher diversity gain performance is associated with a lower ECC value that should be less than 0.5^17^. ECC performance of a MIMO antenna can be calculated through scattering parameters^18^, or through far-field patterns^19^. The following Equation (2) is used to compute ECC using S-parameters

2 $$\begin{aligned} \rho = \frac{\left| S_{11} S_{12}+S_{21} S_{22}\right| ^2}{\left( 1-\left( \left| S_{11}\right| ^2+\left| S_{21} \right| ^2\right) \right) \left( 1-\left( \left| S_{22} \right| ^2+\left| S_{12}\right| ^2 \right) \right) } \end{aligned}$$

ECC can also be calculated using far-field patterns using the following Eq. (3) mentioned in^19^

3 $$\begin{aligned} \rho _e=\frac{\left| \iint _{4 \pi }\left[ \textbf{E}_1(\theta , \phi ) \cdot \textbf{E}_2(\theta , \phi ) d \Omega \right] \right| ^2}{\iint _{4 \pi }\left| \textbf{E}_1(\theta , \phi )\right| ^2 d \Omega \iint _{4 \pi }\left| \textbf{E}_2(\theta , \phi )\right| ^2 d \Omega } \end{aligned}$$

Where the product of the two electric fields on the numerator can be given by Eq. (4).

4 $$\begin{aligned} \textbf{E}_1(\theta , \phi ) \cdot \textbf{E}_2(\theta , \phi )=E_{\theta 1}(\theta , \phi ) E_{\theta 2}^*(\theta , \phi )+E_{\phi 1}(\theta , \phi ) E_{\phi 2}^*(\theta , \phi ) \end{aligned}$$

The following Eq. (5) is used to calculate the diversity gain of a MIMO antennas^19^.

5 $$ Diversity\;Gain(DG) = 10 \times \sqrt {1 - |ECC|^{2} }  $$

Figure 8 displays the simulated and measured ECC and diversity gain performance of the proposed transparent and flexible MIMO antenna using scattering parameters, which achieves an ECC value lower than 0.005 and corresponding value of 10 for DG in the desired operating frequency band. The ECC is also calculated using far-field patterns and its values are 0.107 at 4.5 GHz while its value is 0.157 and 0.163 at 4.41 and 5.56 GHz, respectively at the center frequency and extreme frequencies of the frequency band for $-$10 dB impedance bandwidth. These ECC values are little higher than ECC values calculated through scatting parameters, however, these are still much less than the required minimum value of 0.5 for ECC^17^.

#### Total active reflection coefficient (TARC) and channel capacity loss (CCL)

TARC highlights the importance of resonance frequency stability even in situations where the phase difference between antenna elements varies^17^. According to Eq. (6) below, this factor can be computed by taking the square root of the ratio between the available power minus the radiated power and the total available power.

6 $$\begin{aligned} \Psi _a^t = \sqrt{\frac{(\text {available power} - \text {radiated power})}{\text {available power}}} \end{aligned}$$

In Eqs. (7) and (8), TARC relation^17^ with measured S-Parameters is described as :

7 $$\begin{aligned} \Gamma _a^t = \frac{\sqrt{\sum _{i=1}^N \left| b_i \right| ^2}}{\sqrt{ \sum _{i=1}^N \left| a_i \right| ^2}} \end{aligned}$$

where $b=Sa$ in the above equation.

8 $$\begin{aligned} \Gamma _a^t = \sqrt{\frac{\left| S_{11} +S_{12}e^{j\theta }\right| ^2 + \left| S_{21}+S_{22}e^{j\theta } \right| ^2 }{2}} \end{aligned}$$

where $\theta$ stands for the input feeding phase.

**Figure 9:**
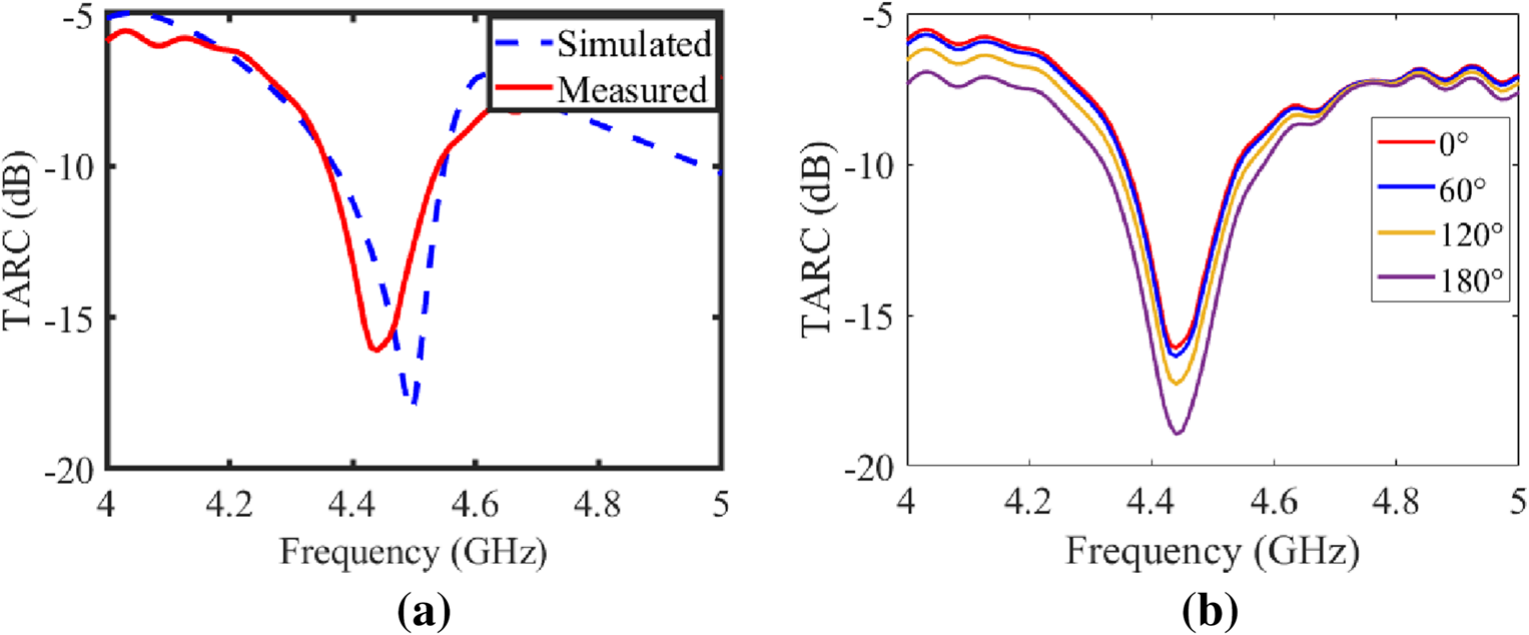
(**a**) Simulated and Measured TARC for port 1 with 0 degree phase angle, (**b**) Measured TARC for port 1 with different phase angles of the counterpart port from 0 to 180 degrees.

Figure 9a presents TARC performance for port 1 of the proposed transparent and flexible MIMO antenna, which is $-$15 dB at 4.5 GHz. TARC is also calculated by considering other excitation vectors with phase variation from 0 to 180 degrees and shown in Fig. 9b. The TARC curve for port 1 remains almost unaffected due to these phase variations. Similar TARC curves are observed for port 2, however, the TARC curves for port 2 are not shown here for brevity.

**Figure 10:**
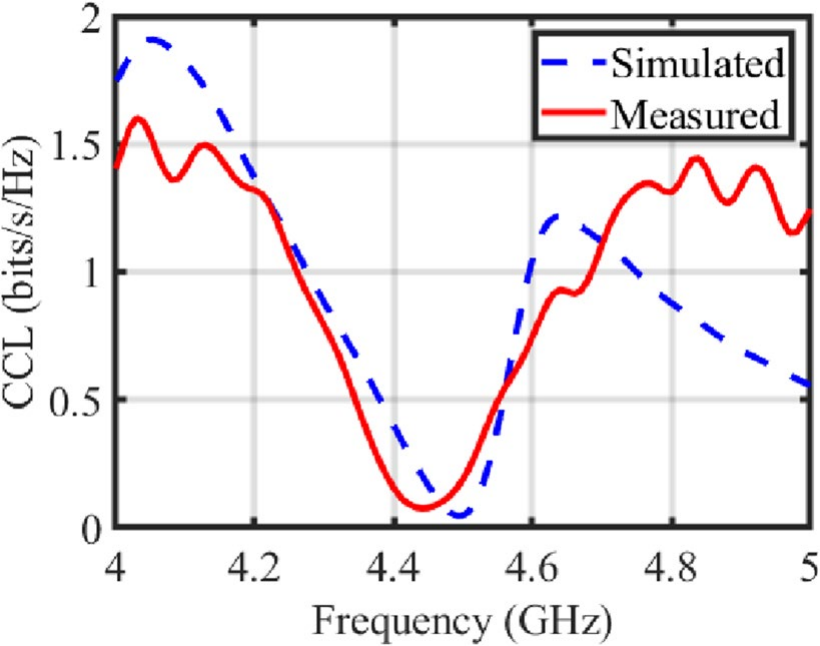
CCL, performance for the proposed transparent and flexible MIMO antenna.

CCL metric is employed to quantify loss for number of data bits per second per Hertz via a communication channel^17^.

Figure 10 depicts the CCL performance of the proposed transparent and flexible MIMO antenna, indicating that the CCL value remains below 0.04 bps/Hz in the desired frequency band. The below Eq. (9) is used to calculate the CCL^17^ parameter:

9 $$\begin{aligned} CCL= - log_2 ^ {|\psi ^\textit{R}|} \end{aligned}$$

where $$|\psi ^\textit{R}|$$ = [$$\rho _{ij}$$],  *(i,j)* $$\epsilon $$ (1,2)

while $\rho _{ij}$ is represented as:

10 $$\begin{aligned} \rho _{11}= & {} {(1-|S_{11}|^2-|S_{12}|^2)} \end{aligned}$$

11 $$\begin{aligned} \rho _{22}= & {} {(1-|S_{21}|^2-|S_{22}|^2)} \end{aligned}$$

12 $$\begin{aligned} \rho _{12}= & {} {(S^*_{11}S_{12} - S^*_{21}S_{22})} \end{aligned}$$

13 $$\begin{aligned} \rho _{21}= & {} {{(S^*_{22}S_{21} + S^*_{12}S_{11})}} \end{aligned}$$

**Table 3:** Comparison with previous recent works.

Ref. no	Freq. bands (GHz)	Substrate	Conductor	Element spacing (mm)	Flexiblity	O.T (%)	Isolation (dB)	Peak gain (dBi)	Radiation eff. (%)
^15^	4.4–5	PET	Ni-metallic mesh	5	Yes	91	20	3.8	85
^16^	2.21–6	Melinex	AgHT-4	11.08	Yes	70	15	0.53	41
^20^	4.67–4.94	Plexi-glass	AgHT-8	5	No	45	10	1.83	53
^21^	2.4–11	Glass	ITO & FTO	20	No	72	20	2	60
**This work**	4.41–4.56	PET	Wired metal mesh	0.9	Yes	83	19	3.2	61

Comparative analysis of the proposed work with previous recent similar works is also presented in Table 3. It can be seen that the proposed transparent and flexible MIMO antenna even with closely spaced antenna elements has demonstrated comparable performance with significantly closed spacing between antenna elements as compared to other similar works.

## Conclusion

A transparent and flexible MIMO antenna design is presented in this study, featuring patch elements in close proximity, which are developed with a wired metal mesh having a square lattice to achieve optical transparency. The substrate material used is a 0.1 mm thick transparent and flexible PET material. To achieve isolation, an offset fed configuration is used, which delivers more than 19 dB measured isolation in the desired frequency band. The proposed MIMO antenna is optimized and analyzed based on the square lattice wired metal mesh parameters and the offset position of the edge fed. The proposed MIMO antenna has achieved 83% transparency and operates at 4.5 GHz. The performance of the proposed transparent and flexible MIMO antenna is evaluated based on parameters such as gain, radiation efficiency, total efficiency, envelope correlation coefficient, diversity gain, TARC, and mean effective gain. The results of the fabricated prototype measurement confirm the suitability of the proposed transparent and flexible MIMO antenna for compact applications requiring optical transparency and visual aesthetic with flexibility. The presented technique could play a crucial role in developing future smart wireless transparent and flexible devices.

## Data Availability

All the data required to evaluate the findings of this work is available in the manuscript. Any other additional data related to this work may be requested from the corresponding author (s).
