# Author Correction: Design and optimization of a transparent and flexible MIMO antenna for compact IoT and 5G applications

**DOI:** 10.1038/s41598-024-52190-5

**Published:** 2024-01-23

**Authors:** Muhammad Nawaz Abbasi, Abdul Aziz, Khaled AlJaloud, Abdul Rehman Chishti, Ali H. Alqahtani, Durria Abbasi, Farooq A. Tahir, Zia Ullah Khan, Rifaqat Hussain

**Affiliations:** 1 Faculty of Engineering & Technology, The Islamia University of Bahawalpur, Bahawalpur, Pakistan; 2 College of Engineering, Muzahimiyah Branch, King Saud University, P.O. Box 2454, Riyadh, 11451 Saudi Arabia; 3 School of Electrical Engineering and Computer Science, National University of Sciences and Technology (NUST), Islamabad, Pakistan; 4 Department of Electronic and Electrical Engineering, The University of Sheffield, Sheffield, UK; 5 Antenna and Electromagnetics Research Group, School of Electronic Engineering and Computer Science, Queen Mary University of London, London, UK

Correction to: *Scientific Reports* 10.1038/s41598-023-47458-1, published online 23 November 2023

The Acknowledgements section in the original version of this Article was omitted. The Acknowledgements section now reads:

“This work was supported by Researchers Supporting Project number (RSP2023R474), King Saud University, Riyadh, Saudi Arabia.”

The original Article has been corrected.

